# Responsivity of Periaqueductal Gray Connectivity Is Related to Headache Frequency in Episodic Migraine

**DOI:** 10.3389/fneur.2018.00061

**Published:** 2018-02-13

**Authors:** Linda Solstrand Dahlberg, Clas N. Linnman, Danielle Lee, Rami Burstein, Lino Becerra, David Borsook

**Affiliations:** ^1^Department of Anesthesiology, Perioperative and Pain Medicine, Boston Children’s Hospital, Harvard Medical School, Boston, MA, United States; ^2^Department of Psychiatry, Massachusetts General Hospital, Harvard Medical School, Boston, MA, United States; ^3^Center for Pain and the Brain, Harvard Medical School, Boston, MA, United States; ^4^Department of Anesthesiology, Beth Israel Deaconess Medical Center, Harvard Medical School, Boston, MA, United States; ^5^Department of Radiology, Massachusetts General Hospital, Harvard Medical School, Boston, MA, United States

**Keywords:** pain, functional connectivity, headache, periaqueductal gray, prefrontal cortex, migraine

## Abstract

Migraineurs show hypersensitivity to sensory stimuli at various stages throughout the migraine cycle. A number of putative processes have been implicated including a dysfunction in the descending pain modulatory system in which the periaqueductal gray (PAG) is considered to play a crucial role. Recurring migraine attacks could progressively perturb this system, lowering the threshold for future attacks, and contribute to disease chronification. Here, we investigated PAG connectivity with other brain regions during a noxious thermal stimulus to determine changes in migraineurs, and associations with migraine frequency. 21 episodic migraine patients and 22 matched controls were included in the study. During functional MRI, a thermode was placed on the subjects’ temple delivering noxious and non-noxious heat stimuli. A psychophysiological interaction (PPI) analysis was carried out to examine pain-induced connectivity of the PAG with other brain regions. The PPI analysis showed increased PAG connectivity with the S1 face representation area and the supplementary motor area, an area involved with pain expectancy, in patients with higher frequency of migraine attacks. PAG connectivity with regions involved with the descending pain modulatory system (i.e., prefrontal cortex) was decreased in the migraineurs versus healthy individuals. Our results suggest that high frequency migraineurs may have diminished resistance to cephalic pain and a less efficient inhibitory pain modulatory response to external stressor (i.e., noxious heat). The findings support the notion that in migraine there is less effective pain modulation (*viz*., decreased pain inhibition or increased pain facilitation), potentially contributing to increased occurrence of attacks/chronification of migraine.

## Introduction

The periaqueductal gray (PAG) modulates signals from deeper brainstem structures and cortical areas, and is implicated in behaviors related to sensory, emotional, autonomic, and motor processes (1, 2). Most of these features are observed in migraine, where sensory (headache, auras, dizziness, photophobia, phonophobia), emotional (anxiety, depression), and autonomic (cardiovascular, nausea, and micturition) problems may be present prior to or during a migraine attack (3, 4). PAG dysfunction has been implicated in a number of chronic pain diseases including migraine (5–9) and altered modulatory function has been considered to be a contributor to these disease entities. Given that PAG neurons [which may be inhibitory or facilitory (10, 11)] send projections to the dorsal horn (spinal cord) or trigeminal nucleus *via* the rostral ventromedial medulla (12), the overall efferent output from the PAG may inhibit or facilitate afferent trigeminal sensory (pain) inputs *via* the raphe magnus (13) to the second order neurons in the trigeminal sensory complex (1, 14, 15) that project rostrally to thalamus and other subcortical regions. Altered efficiency (i.e., deficiency in inhibitory or excessive facilitory outputs) along this pathway or in modulating brainstem structures may lead to altered function and contribute to increased migraine intensity and diminished resistance to migraine attacks (7, 16). Frequency of attacks may be influenced by alterations in PAG tone (16) and the overall diminished resistance to attacks may contribute to more significant changes previously observed in imaging studies of brain structure and function (17–20).

Pain changes the functional connectivity (Fc) between the PAG and other brain regions (21), but less is known about the effect of pain perturbation on PAG connectivity in migraine, and potential relations to migraine disease burden. Using the PAG as a region of interest (ROI), we evaluated differences in Fc between migraineurs and healthy controls (HCs) during noxious heat stimulation applied to the V1 region of the face. We further examined how such pain-induced PAG connectivity may relate to migraine frequency. The difference in connectivity between the PAG and other brain areas—in episodic migraineurs and age-gender matched controls—was examined using a psychophysiological interaction (PPI) analysis. A PPI analysis tests how much of the variance of BOLD signal can be explained by the interaction between signal in one seed ROI (the physiological parameter, here the PAG) and an experimental variable (the psychological variable, here a noxious heat stimulus to the temple). We hypothesized that facial pain stimulation might lead to differentiated patterns of PAG Fc between migraineurs and controls. The rationale for this is that repeated headache attacks (increased pain load) would lead to alterations in endogenous inhibitory modulatory state. Further, we also hypothesized that migraine frequency would influence the degree of connectivity between the PAG and pain modulatory regions. Similar evaluation of an individuals PAG neural dynamic responsivity to pain may in the future contribute to defining treatment effects and predicting outcomes since some therapeutic changes may be incremental and progressive.

## Materials and Methods

### Subjects

Twenty-one migraine patients fulfilling the criteria of the International Classification for Headache for episodic migraine were recruited for this study. All patients had suffered migraines for more than 3 years. The imaging sessions took place during the migraineurs’ interictal phase, when a migraine attack had been absent for at least 48 h, and no attacks occurred the next 24 h after the scan. The patient’s migraine frequency was recorded based on a questionnaire and a migraine diary. Twenty-two HCs were included in the study and included and matched with the patients based on gender and age. Exclusion criteria for the HCs included a history of psychiatric or neurological disorders, chronic pain, or other major diseases, as well as any significant history of pain. Beck Depression Inventory II (22) was used to screen subjects for depression, with an exclusion set to 25 points of more, indicating moderate to severe depression. A urine sample was obtained from all subjects to test for any use of benzodiazepines, cocaine, amphetamine, phencyclidine, barbiturates, tetrahydrocannabinol, or non-prescription opioids. The institutional review board at McLean Hospital approved the study, which was carried out in adherence to the standards set by the Declaration of Helsinki. After written consent was obtained, the subjects were scanned at McLean Hospital.

### Heat Stimulation

All subjects received thermal stimulation through a 3 cm × 3 cm thermode (TSA-II, Medoc Advanced Medical Systems) placed on the temple. The dominant migraine side was used for stimulation in the migraineurs, meaning 6 out of 21 migraineurs were stimulated on the right side of the head. Where no migraine laterality was reported (*n* = 9), the left side was used as the default thermode site. Thermode placement side for the HCs was corresponding to their matched migraine patient. Stimulation levels need to induce moderate pain were determined in the following way; from a baseline temperature of 32°C, temperature was increased by 1°C per second until the temperature resulted in a pain rating of 7 on a 10 point scale with 10 meaning the worst possible pain, and 0 being no pain. Three trials were used and the average temperature needed to induce a “7” was then used as the moderate pain intensity stimulus in the scan sessions.

### Scanning Paradigm

During the scan, the subjects were initially presented with a 55-s baseline of non-painful stimulation (32°C). Following were five cycles of thermal stimulation where the temperature of the thermode increased until the individual predefined 7/10 pain threshold was reached. The stimulation lasted for 15 s before the temperature decreased to baseline, with an interstimulus interval of 30 s.

### Image Acquisition

A 3 T Siemens Trio scanner was used to obtain both functional and structural images with a 12-channel headcoil. Functional images from the heat paradigm were obtained with a gradient echo–echo planar pulse sequence (GE-EPI) with a 3.5 mm × 3.5 mm × 3.5 mm voxel resolution. Scanning parameters for fMRI data included: Repetition Time (TR) = 2,500 ms, time of echo (TE) = 30 ms, Field of View = 224 mm^2^, Flip Angle (FA) = 90^o^, slices = 41 (in axial orientation), volumes = 256, acquisition time = 8:40 min.

The structural T1 weighted imaging was carried out with a magnetization-prepared rapid acquisition gradient-echo sequence with the following parameters: TR = 2,000 ms, TE = 3.53 ms, time of inversion = 1,100 ms, FA = 8^o^, slices = 224 (in sagittal orientation), acquisition time = 3:36 min.

### Preprocessing Pipeline for Functional Paradigm

Preprocessing and analysis of functional data was performed with FEAT (FMRI Expert Analysis Tool) Version 6.00, a function of FSL (FMRIB’s Software Library, www.fmrib.ox.ac.uk/fsl). Motion correction was carried out with the Linear Motion Correction tool (MCFLIRT); Fourier-space time series phase-shifting for interleaved slice acquisition to perform slice-timing correction; brain extraction tool was used to extract the brain from non-brain tissues; spatial smoothing was carried out with full width, half maximum of 5 mm; normalization of the grand-mean intensity was performed on the 4D; and a high-pass filter with a 80-s cutoff was used for temporal filtering. To reach signal equilibrium, three dummy scans were collected at the start of the scan and discarded. The functional images of the six subjects who were stimulated on the right-hand side of the head were right-left flipped before continuing further analysis. Co-registration of functional data to the corresponding high-resolution structural image was carried out before being registered to the MNI152 template brain with the Linear Image Registration tool (FLIRT).

### Statistical Analysis

#### First-Level Analysis

The individual subjects’ time series were analyzed using FMRIB’s Improved Linear Modeling utilizing the local autocorrection method in FSL (23). Onsets (excluding ramp-up and ramp-down) for the thermal stimuli from each subjects’ temperature recordings were inserted as explanatory variables (EVs), and convoluted using a gamma hemodynamic response function. A PPI analysis was carried out in order to examine the Fc from the PAG to other regions in the brain during the pain stimulation. This was done by extracting the time series from a manually masked ROI (the PAG) during the experimental paradigm (painful, non-painful, and baseline blocks). The interaction between the physiological region (PAG) and the psychological condition (i.e., pain) is then introduced as a regressor in the analysis. This EV, containing the task dependent PAG activity is correlated with the activity in other voxels in the brain. Other EVs consisted of the main effects of painful and non-painful stimulation and the PAG time-course. Motion parameters in six dimensions, spikes in signal as well as white matter and CSF signal were included as confounding variables.

#### Second-Level Analysis

Group differences in pain-induced connectivity from the PAG, as well as a correlation covariate with migraine frequency and PAG pain-induced connectivity, were tested using FMRIB’s Local Analysis of Mixed Effects in FEAT. Instead of using an arbitrary *z*-statistic threshold for activated clusters as is typically done in null hypothesis testing, the statistical maps were investigated with a mixture model (mm) that determines significant activation and deactivation based on the data. In other words, the model examines if the activation is larger than the overall activation of all voxels, instead of testing whether the activation is different to 0 activation. In this way, a more objective inference of activation can be made, rather than using a random threshold as a marker of elevated activation. Thus, *z*-statistical maps were included in a spatial mm, carried out with FSL’s mm (24). By using the default threshold of 0.5, the model will produce probability maps of voxels belonging to either the “no activation” (noise), “activation,” or “deactivation” where the probability of the classification being a false positive or a false negative is equal (50%). The resulting activity maps where then corrected with a volume extent threshold of 20 voxels. The mm also makes assumptions that there is activation in proximity to other activated areas, and in that way spatial parameters are adapted objectively from the data. Because mm reports clusters that survive the classification threshold of 0.5, coordinates of the center of gravity of each voxel is reported instead of the local maxima. Significant clusters were identified by the anatomical automatic labeling atlas in the WFU pickatlas toolbox in SPM (25), and by using anatomical landmarks.

Since one of the migraine patients used a migraine preventative drug (topiramate) which is found to alter cortical excitability (26), we performed the analyses with and without this subject including a covariate in the analysis to regress out the effect of this drug.

## Results

### Behavioral Data

16 females and 5 males were included in the migraine group (average age: 32.71 ± 8.3 years), and 17 females and 5 males were included in the control group (average age: 32.96 ± 8.9 years). The average duration of illness in the migraine group was 15.8 ± 11.8 years, and average monthly frequency of attacks was 6.3 ± 5.3. See Table 1 for descriptive data. Temperatures rated as moderately painful [7/10 on a visual analog (VAS) scale] delivered by the thermode to the V1 region (forehead) were similar between migraineurs and HCs (migraine: 46.7 ± 2.03°C; HCs: 46.87 ± 1.48°C, *p* = 0.756).

**Table 1:** Clinical and descriptive data of migraine patients.

Subject	Age	Migraine frequency (per month)	Condition duration (years)	Medications for migraine
1	36–40	2	20	Aspirin, tylenol, ibuprofen
2	31–35	15	14	Excedrin
3	21–25	6	8	Tenormin, tylenol, aleve, maxalt
4	21–25	12	7	Tylenol, ibuprofen
5	21–25	20	7	Ibuprofen
6	21–25	1	3	Caffeine + acetaminophin
7	31–35	12–13	31	Aspirin, ibuprofen
8	36–40	2–3	33	
9	21–25	1	4	Tylenol
10	26–30	10	3	
11	36–40	8	28	
12	26–30	1–3	9	
13	46–50	2–5	31	Sumatriptan, ibuprofen
14	36–40	4	27	Topamax, ibuprofen, aspirin
15	36–40	2–3	12	
16	31–35	2–3	26	Tylenol
17	41–45	1–2	25	
18	41–45	3–4	23	Ibuprofen
19	26–30	2–4	12	
20	46–50	11	30	Excedrin for migraines
21	21–25	7–10	3	

### Psychophysiological Interaction

**Figure 1:**
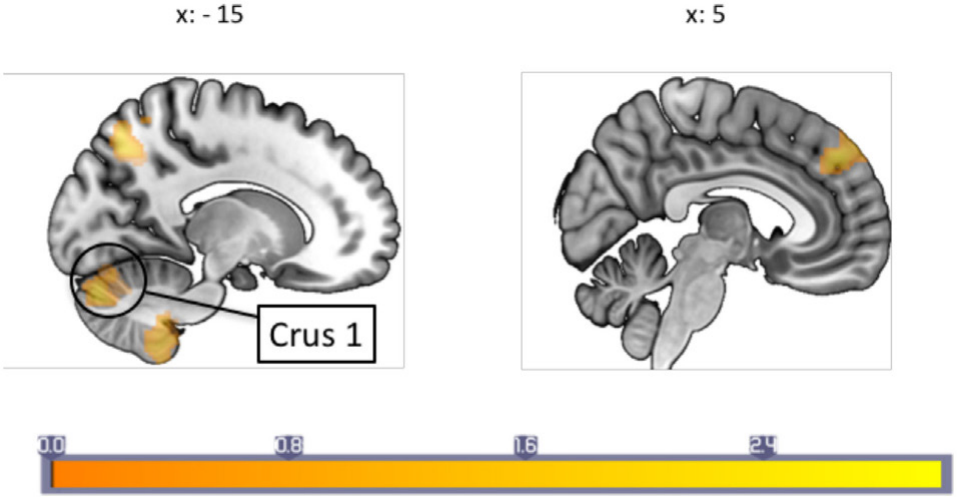
A between-groups comparison of periaqueductal gray connectivity during painful stimulation showed that healthy controls had increased connectivity with the superior prefrontal cortex, and cerebellum crus I among other regions not shown in image. Each sagittal slice is numbered with the respective Montreal Neurological Institute *x*-coordinate. Color bar indicates *z*-statistic.

The PPI analysis using the PAG as the physiological seed region, and pain as the psychological variable showed a main effect of pain-induced PAG connectivity with a cluster over the left anterior cingulate cortex (ACC), the right cerebellum crus I, the vermis, the right fusiform gyrus as well as the right middle temporal lobe, whereas a negative main effect was seen in the pallidum, supplementary motor area (SMA), cerebellum, pre- and postcentral gyrus, among other areas (see Table S1 in Supplementary Material for a full list). In between-groups contrasts, the migraine group revealed increased pain-related Fc to the SMA, sensorimotor cortex face area, the insula, bilateral thalamus, and pons (see Table 2 for a full list) when contrasting the migraine with the control group. Controls, as compared to migraineurs had stronger pain-induced connectivity to the superior medial frontal cortex, the cerebellum (lobule IX and crus I), the middle occipital gyrus, the temporal pole, as well as the superior parietal cortex (see Figure 1). The overall findings were still present when the subject using topiramate was excludedmedicine covariate was introduced, but the PAG–superior frontal cortex connectivity was no longer significant in the HC–migraine contrast.

**Table 2:** A psychophysiological interaction (PPI) analysis revealed differences in pain-induced connectivity from the periaqueductal gray in migraineurs and controls.

			COG MNI coordinates			
Brain region	Laterality	*Z*-Stat	*X*	*Y*	*Z*	Vol (voxels)
**HC > migraine**						
Temporal pole sup	L	2.73	−46	20	−20	704
Precuneus	L	2.56	−12	−68	52	482
Front Sup Medial	R	2.38	6	50	40	478
Cerebellum IX	L	2.5	−20	−46	−50	450
Cerebellum Crus 1	L	2.47	−18	−78	−28	422
Mid. Occipital	R	2.89	30	−90	18	267
Cerebellum Crus 1	R	2.45	30	−86	−24	207
Mid. Occipital	L	2.51	−30	−98	−4	179

**Migraine > HC**						
Calcarine	R	2.74	10	−78	14	2,977
Postcentral	L	2.43	−60	−14	28	2,010
SMA	R	2.62	12	−16	70	1,424
Insula	R	2.39	42	−6	10	405
Precentral	R	1.96	42	−10	50	373
Sup. occipital	L	2.27	−20	−84	44	368
Middle temporal	R	2.25	52	−58	8	353
Inf. orbital	L	2.15	−38	34	−12	192
Precentral	L	2.26	−24	−24	72	187
Fusiform gyrus	L	2.12	−32	−24	−30	165
Parahippicampal area	R	2.19	28	−12	−26	155
Thalamus	L	2.53	−10	−12	−2	131
Angular gyrus	L	2.48	−48	−74	32	123
Cuneus	L	1.72	−10	−72	28	120
Thalamus	R	2.14	10	−20	4	93
Medial orbital	R	1.71	4	62	−14	84
Postcentral	L	2.44	−22	−36	58	79
Pyramis	L	2.04	−46	−78	−44	44
Fusiform gyrus	R	1.81	28	−72	−8	21

*COG, center of gravity; MNI, Montreal Neurological Institute coordinates; SMA, supplementary motor area; Vol, volume*.

#### Relation to Migraine Frequency

**Figure 2:**
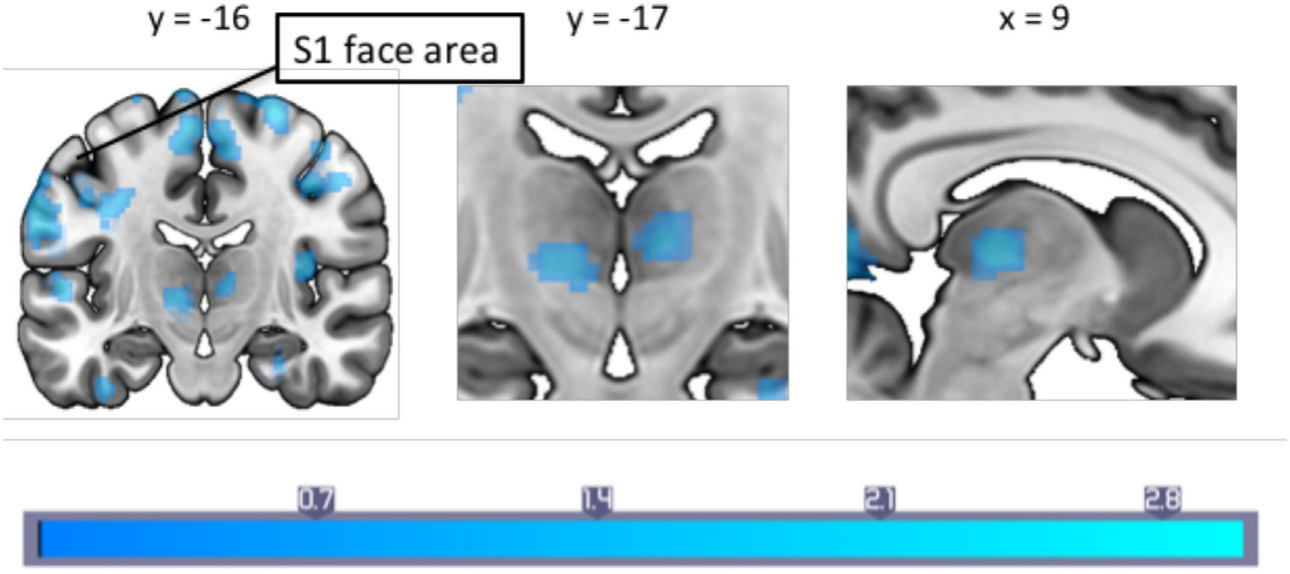
A between-groups comparison of pain-induced periaqueductal gray connectivity showed that in comparison to healthy controls, migraineurs had increased connectivity with the bilateral thalamus, supplementary motor area and the primary somatosensory cortex face area. Montreal Neurological Institute coordinates are marked in the respective dimensions. Color bar indicates *z*-statistic.

**Figure 3:**
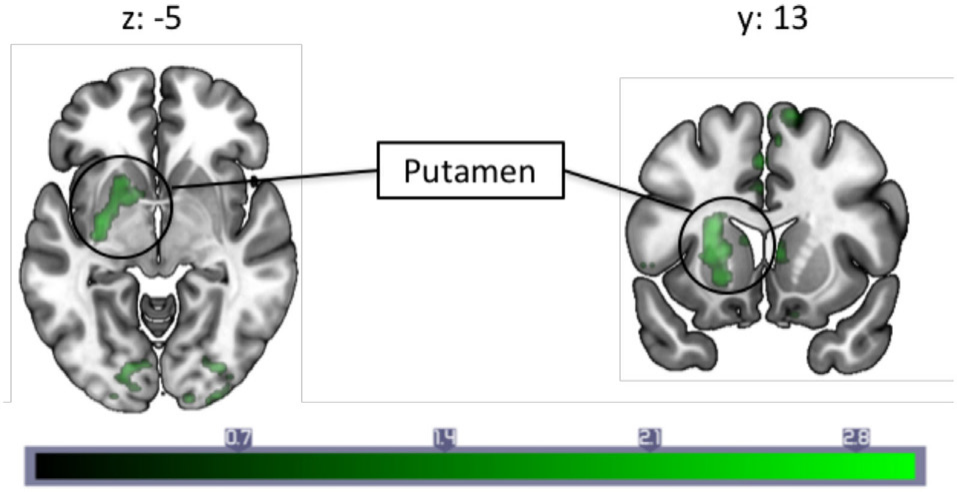
A correlation analysis with monthly migraine frequencies and pain-induced periaqueductal gray connectivity revealed a negative correlation with connectivity to the left putamen. Each slice is numbered with the respective Montreal Neurological Institute coordinate. Color bar indicates *z*-statistic.

A correlation with migraine frequency revealed that the more frequent migraines, the higher the connectivity from the PAG to the SMA, an orbitofrontal cluster including the caudate, and the precentral gyrus face area (Figure 2, see Table 3 for a full list). Several regions showing pain-induced connectivity with the PAG were found to be negatively correlated with migraine frequency, including the putamen, superior frontal gyrus, the SMA, and the ACC (see Figure 3). Coordinates and volumes of each region found to correlate significantly with the frequency of migraine attacks are reported in Table 3. The correlations persisted, albeit at slightly lower significance levels, when the subject on topiramate was excluded.

**Table 3:** A correlation analysis revealed a correlation between monthly frequencies of attacks with periaqueductal gray connectivity strength during painful stimulation.

			COG MNI coordinates			
Brain region	Side	*Z*-stat	*X*	*Y*	*Z*	Vol (voxels)

Frequently correlation						
**Positive correlation**						
WM (cluster including orbitofrontal cortex and caudate)	L	2.62	−24	34	−6	2,894
Sup. occipital	R	2.58	24	−70	30	2,258
SMA	R	2.83	4	−18	70	1,847
Cerebellum 9	R	2.56	8	−48	−58	941
Inf. frontal	R	2.43	36	18	34	861
Precentral/postcentral (face area)	L	2.27	−56	−8	32	683
Cerebellum crus 1	R	2.42	46	−62	−40	578
Inf. Temporal gyrus	R	1.97	52	0	−40	284

**Negative correlation**						
Putamen	L	2.5	−22	4	6	871
Sup. frontal	R	2.58	20	48	40	334
Sup. parietal	R	2.33	26	−62	60	273
Precuneus	L	2.07	−8	−68	58	221
Cerebellum VI	R	2.09	18	−68	−28	178
Cerebellum VIII	R	2.1	28	−42	−52	159
Sup. temporal lobe	L	2.42	−48	−20	12	154
SMA	R	2.51	4	8	68	150
Mid. occipital	R	2.12	32	−94	2	146
Mid. cingulum	L	2.23	45	77	52	141
Precentral	L	2.2	−26	−14	56	118
Sup. occipital	R	2.55	28	−92	22	107
Cerebellum 9	L	2.28	−22	−42	−48	99
Precentral	L	2.38	−38	2	60	83
Inf. frontal tri	L	2.07	−54	32	14	80
Mid. frontal	L	2.5	−40	44	30	76
Cerebellum IV/V	R	2.17	18	−52	−20	71
Paracentral lobule	L	1.85	−14	−36	74	60
Precentral	R	2.1	30	−26	72	60
Insula	L	2.15	−38	−4	16	60
SMA	L	2.02	2	4	40	58
Cerebellum VIII	L	1.73	−44	−48	−50	55
Cerebellum crus 2	L	2.08	−26	−78	−46	54
Temporal pole	L	2.24	70	74	29	52
Precentral	L	1.99	−32	−30	40	51
ACC	R	2.37	4	34	10	50
Vermis 4/5		1.99	2	−50	-22	47
Caudate	R	2.2	6	12	6	41
SMA	L	2.2	−2	12	48	34
Mid. frontal	R	2.18	30	30	28	34
Precentral	R	1.94	48	6	28	30
Sup. frontal	L	2.36	−26	46	40	29
Lingual gyrus	R	2.06	18	−100	−8	28

*ACC, anterior cingulate cortex; COG, center of gravity; Inf, inferior; Mid, middle; MNI, Montreal Neurological Institute coordinates; SMA, supplementary motor area; Sup, superior; Vol, volume; WM, white matter*.

## Discussion

Our study examined PAG connectivity during a pain stressor in episodic migraine patients during their interictal period. Across subjects, painful stimulation led to an increase in PAG Fc to the right ACC, cerebellum crus I, and the middle temporal gyrus, and decreased connectivity to the right SMA, left pallidum, the pre- and postcentral gyrus, among others. Due to the large number of regions found to be differentially functionally connected in migraine in the current study, we focus below on those that have known structural and/or Fc to the PAG. As reviewed in Linnman et al. (2), connections to the PAG have primarily been described encompassing a medial limbic network and a lateral sensory network, as evidenced by DTI, fMRI and tracing studies.

When contrasting migraine subjects versus HCs, migraine subjects had a lower degree of pain-induced PAG connectivity to regions that are typically associated with pain modulation, whereas an increased connectivity was seen to sensory regions (i.e., the SMA, sensorimotor cortex, thalamus, and insula). Furthermore, our results indicate that painful stimulation in patients with a higher frequency of migraine attacks is linked to increased PAG connectivity with the primary somatosensory cortex region representing the face area, and the SMA. This increased connectivity may be a consequence of repeated (frequency related) engagement of these connections and the potential changes in S1 are reflected in our prior studies of both children and adults using arterial spin labeling (27, 28). The current findings suggest that, as a consequence to a dysfunctional pain modulatory system, patients with migraine fail to fully engage this system to inhibit pain, even though overt behavioral manifestations may only be evident during more intense stimuli. Taken together, the results suggest the descending pain modulation system is compromised and may contribute to added allostatic load (*viz*., increased migraine attacks) because pain modulatory regions are less effective in inhibiting trigeminal afferent inputs (16).

### Pain-Induced PAG Connectivity

During experimental heat pain, healthy individuals, in comparison to interictal migraine patients showed stronger PAG connectivity with superior medial prefrontal cortex, the cerebellum, middle occipital gyrus, the temporal pole, and the precuneus. The prefrontal cortex has been shown to be involved in descending pain modulation (29–32), a function that may be impaired in episodic migraineurs. In addition to other regions, migraineurs compared to controls showed stronger pain-induced connectivity with the SMA, sensorimotor cortex, and thalamus—regions that activate in response to nociception and anticipation to pain suggesting a hypersensitive pain pathway in migraineurs (33, 34). The observed changes in cortical (PFC, SI, SMA), subcortical (thalamus, putamen, and cerebellum) regions are discussed below.

### PAG Fc and the PFC/ACC—Altered Descending Modulatory Pathways

The PFC has projections to the PAG and a number of fMRI studies have shown altered Fc in clinical pain conditions (8, 9, 35). Reduced communication (i.e., decreased Fc) between the PAG and PFC and by inference, descending modulatory regions (36), during noxious heat stimulation was observed. However, this finding should be interpreted with caution, as this finding was no longer significant when the migraine patient using topiramate was excluded from the analysis. Nonetheless, we interpret these findings as diminished responsiveness of inhibitory modulatory systems and suggest that as a consequence, repeated afferent pain signals during a migraine attack contribute to a diminished allostatic state. This is supported by our finding that the PAG and superior frontal cortex [and the ACC, another major component in descending modulatory pain system (8, 9, 35)] connectivity is perhaps more effective in those with a low frequency of attacks as revealed by the correlation analysis, but that this function may be exacerbated after repeated engagement in high frequency migraineurs. Further support for our findings come from resting-state studies: the ventrolateral PAG showed decreased connectivity with the medial PFC, ACC, and orbitofrontal cortex during rest, where connectivity between these areas decreased as migraine frequency increased (5); and decreased PAG connectivity to the PFC and the ACC that was also associated with an increase in migraine frequency in another study (37). These resting-state studies suggest a weakening efficacy of the descending pain modulatory pathway in migraine patients similar to the results presented here. Whether worsening of migraine frequency further perturb this system or is a consequence thereof remains to be examined.

### PAG Fc and Primary Somatosensory Region (S1)

We observed a higher PPI effect in migraineurs with regards to pain-induced connectivity between the PAG and the ipsilateral S1 face area. Further, a correlation between pain-induced PAG connectivity and the frequency of migraine attacks revealed that migraineurs with a higher frequency of attacks had increased connectivity between the PAG and the ipsilateral S1 and M1 face representation area during painful stimulation. As the heat probe was on the temple, the nociceptive signal engaged the trigeminal nerve, and the result likely reflects sensitization of the trigeminal nerve pathway. One possible explanation is that this reflects increased pain facilitation resulting in high frequency migraineurs to develop cephalic allodynia, thereby contributing to lowering the threshold for reoccurring migraine attacks. Even though this theory is not supported by the current findings, it lends support from a previous study that reported long-lasting cephalic cutaneous hypersensitivity and sensitization of the trigeminal nerve following frequent high-intensity stimulation of the dura in rats (38). Even though the temperature thresholds and reported pain ratings on the VAS-scale of the heat stimulation did not differ between the two groups in our study, others have reported allodynia in the interictal period of migraineurs (39). The idea of sub-allodynia is discussed in a previous study where migraineurs in the interictal period were exposed to a repeated painful stimuli but pain thresholds did not reach a “clinical expression” (40). Repeated exposure to such stressors lowers cutaneous perception in migraineurs. Our results add to the accumulating evidence that increased migraine attacks may reflect central sensitization in combination with increased pain facilitation, perhaps leading to migraine chronification (38, 41, 42). We provide findings that indicate direct communication between the PAG and the sensorimotor cortex face representation area (*via* the trigemino-thalamic-cortical pathway) may be involved in this process. Notably, the effect was on the ipsilateral side of stimulation. Henssen et al. recently proposed the co-existence of an ipsilateral nociceptive conduction tract to the cerebral cortex (43), with ipsilateral sensory projections *via* the dorsal primary sensory nucleus, and ipsilateral PAG modulation *via* the caudal part of the spinal nucleus. Given our results, this potential ipsilateral pathway may have precedence in migraine.

### PAG Fc and the SMA—Pain Anticipation in Migraineurs

A basis for altered expectancy in migraineurs has been reviewed elsewhere (44). PAG connectivity in migraine patients in comparison to HC, as well as the frequency correlation showed increased connectivity with the SMA. The SMA is well known for being involved with motor planning and preparation (45); however, the SMA is also found to be activated during anticipation of a painful stimulus (46, 47), and it is also reportedly active during pain that is perceived as uncontrollable (48). It is likely that migraineurs with high frequency of migraines commonly engage this region in anticipation of the next attack, a painful stressor that is uncontrollable. Moreover, structural differences in the SMA are found to correlate with helplessness in chronic pain patients, a population that is at higher risk of developing learnt helplessness due to the prolonged exposure of an unavoidable aversive stimulus (pain) (49). Aberrant SMA functioning in migraine is supported by previous imaging studies that have reported an increased likelihood of SMA activation in female migraineurs in most frequency bands as measured by magnetoencephalography while making movements during migraine (50). Decreased regional homogeneity values in the SMA have been found in migraineurs compared to the healthy (51), and migraineurs show a displacement of the SMA compared to HCs (52). Despite the frequency of appearance of the SMA in migraine studies, it is often overlooked. These findings in combination with our current results suggest that the SMA may play a bigger role in migraine chronification than considered previously, perhaps pertaining to migraine expectancy and helplessness in the face of an inescapable stressor that is dependent on communication with the PAG.

### PAG Fc and the Putamen

A negative correlation between migraine frequency and pain-induced connectivity was observed in the putamen, a part of the basal ganglia, meaning this region has increased connectivity in migraineurs with less frequent migraine attacks. The basal ganglia, including the putamen, are implicated in numerous functions, including pain processing (53, 54). Our lab has previously reported findings on abnormal activation in the basal ganglia (including caudate, putamen, accumbens) in migraineurs compared to controls (55), and similar to the present study, a decreased engagement of the basal ganglia is found in high frequency compared to low frequency migraineurs in response to pain (56). Disease duration has also been found to correlate with PAG–putamen connectivity in migraineurs (57). Furthermore, the putamen is involved in pain and analgesia [for a review, see Ref. (58).]. The change in PAG connectivity with this region as migraine frequency increases could, therefore, indicate (a) the endogenous analgesic response may be more intact with fewer frequencies of attacks, but wears off in tandem with an increased frequency of attacks and (b) since the putamen is found to be activated in chronic and acute pain, a decrease in PAG–putamen connectivity during pain in those with higher frequency of migraine attacks could reflect a pain response that is exacerbated with higher migraine frequency, reflecting habituation or dysfunction. In sum, the decreased PAG–putamen connectivity could be another manifestation of an impaired pain modulatory system in migraineurs.

### PAG Fc and the Thalamus—Hypersensitization of Pain Pathways

Migraineurs in our sample displayed stronger pain-induced connectivity between the PAG and bilateral thalamus. The PAG has bilateral ascending projections to the thalamus (1, 32, 59–61) that activates in response to a pain stressor (21, 61, 62). Our results confirm two prior reports: in the first, increased resting-state Fc between the PAG and thalamus in migraine patients was reported, in particular in patients with severe allodynia (63); in the second, strength of connectivity between these two structures in an episodic migraine population was dependent on disease duration (57). Moreover, one study reported greater resting-state Fc between the PAG and the thalamus was reported (in addition to the insula, somatosensory cortex, angular gyrus, and posterior parietal cortex—all areas that we found to have a pain-induced connectivity in migraineurs when compared to controls) (37). The latter authors suggest that these connections might be due to a hypersensitized pain pathway that is instrumental in migraine pathology (37). As such repeated migraine attacks may exacebate trigeminovascular pathway hyperexcitablitily reflecting facilitation or lack of inhibition of these neurons through PAG related descending modulatory controls (33, 64).

### PAG Fc and Cerebellum: An Area of Interest in Migraine

During painful stimuli, the HC group showed stronger pain-induced PAG-cerebellum connectivity to the bilateral crus I as well as left cerebellar lobule IX than the migraine patients. The role of the cerebellum in sensory processing including pain is still not well defined (65), but seems to play a role in hemiplegic migraine (66, 67). Animal studies indicate that the cerebellum is involved with nociceptive modulation (68, 69), and likely also pain processing (65); therefore, cerebellar involvement in our case could reflect its involvement in a pain modulatory system that is less intact in migraineurs. In humans is the cerebellum now well described to have a role pain processing; a previous study in our lab found that crus I was activated in response to a noxious heat stimuli (70), and another study reported crus I activation in response to trigeminal nociception where connectivity with both the PAG and thalamus was seen to be influenced by pain intensity (71). Additionally, crus I has been found to be implicated in emotional processing (72). Altogether, PAG–crus I connectivity during painful heat stimuli in our sample could, therefore, be yet another indication of dysfunctional processing/modulation of a noxious stimuli that even demonstrates an impaired affective evaluation of the pain stimuli in migraine patients, perhaps due to habituation as has been proposed for other chronic pain conditions (73–75).

### Repeated Pain, Stress, and Allostasis in Migraine

It has previously been argued that repeated migraine attacks as a stressor can alter brain systems and contribute to chronification of the disease (76). The PAG is an important structure in processing internal and external stressors (1). Increase in migraine frequency and severity add to the allostatic load, diminishing the brains’ responses to pain that consequentially impairs the brains’ ability to cope with stressors. Stress levels are repeatedly found to be higher in migraine patients and considered a defining factor in duration and frequency of migraine (77–79). Thus, the altered connectivity in coping response (modulation) to the heat stressor reported here might reflect a system perturbed by excessive internal as well as external stress in patients with a higher frequency of migraine, which potentially may contribute to a chronic migraine state (80). Our findings that migraineurs show decreased PAG connectivity with pain modulation regions (PFC, cerebellum), but increased connectivity with sensory regions (sensorimotor cortex, insula, SMA and thalamus) could, therefore, indicate that pain sensory pathways in migraine are more easily triggered, perhaps aggravated by repeated episodes (as indicated by the frequency correlation), whereas pain modulation pathways are less easy to engage.

### Caveats

As with most studies there are caveats related to this one including: (1) *Thresholding and interference*: spatial thresholding and *p*-value cutoffs are commonly used correction methods in neuroimaging with the aim to avoid type I errors. Most often this threshold is an arbitrary number of voxels where the researchers deem clusters smaller than said number to likely have occurred due to chance, and corrected or uncorrected *p*-value cutoffs based on convention. However, these approaches might not be appropriate with all analyses. We opted for mixture modeling as an unbiased, objective method to infer regions that show pain-induced Fc to the PAG. In contrast to other models such as the general linear model that makes assumptions about the null activation, the mm explicitly models the null activity in addition to the activation and deactivation. Many of our findings in the current study are consistent with previously published literature, and we also provide novel findings highlighting the role of the PAG in migraineurs. (2) *Medications*: most fMRI migraine studies faces the issue of potential confounds of long-term use of medication on brain activity. However, the type of migraine medication used was relatively diverse in the current sample (see Table S1 in Supplementary Material) and is thus not likely to be a determining systematic factor of our results. There is some evidence that the drug topamax (topiramate) may affect cortical excitability (26). One of the migraine subjects in our study was on Topamax at the time of the study. We, therefore, ran the analysis both with and without this subject. The only remarkable difference in the results was the lack of PAG–PFC connectivity when comparing HC with migraineurs. Thus, the results should be interpreted with caution. However, a general reduction in descending pain modulation is still supported by our finding that the high frequency migraineurs had reduced connectivity with both the PFC and the ACC, suggesting dysfunctional pain modulatory system in migraine. (3) *Sex distribution*: another potential confound is the unevenly represented number of female participants in this study. It is widely known that migraine prevalence is higher in females than males, making a balanced sample difficult to recruit. However, our control sample was both sex and age- matched, avoiding confounding effects of sex in our results.

## Conclusion

Using non-invasive imaging methods, we have examined PAG Fc (Fc) during facial pain in episodic migraineurs, and demonstrated found indications of a perturbed system either caused by or influencing migraine frequency. We report new data on a direct relationship between the PAG and the face representation area in S1 with migraine as related to migraine frequency. Moreover, we also provide evidence for pain-induced connectivity with the SMA, an area involved with pain expectancy, as well as an impaired pain modulatory system in migraineurs compared to control. Our findings emphasize the role of the PAG in migraine pathology.

## Ethics Statement

The institutional review board at McLean Hospital approved the study, which was carried out in adherence to the standards set by the Declaration of Helsinki. After written consent was obtained, the subjects were scanned at McLean Hospital.

## Author Contributions

The specific contributions of each author are as follows: LD contributed in analyses, interpretation, and drafted the work; CL advised in analyses, contributed to interpretation and drafting of the work; DL contributed in analyses and drafting of the paper; RB contributed in conception and design as well as interpretation and revising the draft critically; LB contributed in conception and design, advised on analyses, and contributed to drafting the work; DB contributed in conception and design and in interpretation and drafting of the work. All authors have read and approved the final draft of the paper, and agree to accountability of the work that has been conducted.

## Conflict of Interest Statement

The authors declare that the research was conducted in the absence of any commercial or financial relationships that could be construed as a potential conflict of interest.

## References

[1] Benarroch EE Periaqueductal gray: an interface for behavioral control. Neurology (2012) 78:210–7. 10.1212/WNL.0b013e31823fcdee 22249496

[2] Linnman C Moulton EA Barmettler G Becerra L Borsook D. Neuroimaging of the periaqueductal gray: state of the field. Neuroimage (2012) 60:505–22. 10.1016/j.neuroimage.2011.11.095 22197740 PMC3288184

[3] Black AK Fulwiler JC Smitherman TA. The role of fear of pain in headache. Headache (2015) 55:669–79. 10.1111/head.12561 25903510

[4] Silberstein SD. Migraine symptoms: results of a survey of self-reported migraineurs. Headache (1995) 35:387–96. 10.1111/j.1526-4610.1995.hed3507387.x 7672955

[5] Li Z Liu M Lan L Zeng F Makris N Liang Y Altered periaqueductal gray resting state functional connectivity in migraine and the modulation effect of treatment. Sci Rep (2016) 6:20298. 10.1038/srep20298 26839078 PMC4738255

[6] Ho Y-C Cheng J-K Chiou L-C. Hypofunction of glutamatergic neurotransmission in the periaqueductal gray contributes to nerve-injury-induced neuropathic pain. J Neurosci (2013) 33:7825–36. 10.1523/JNEUROSCI.5583-12.2013 23637174 PMC6618956

[7] Welch KM Nagesh V Aurora SK Gelman N. Periaqueductal gray matter dysfunction in migraine: cause or the burden of illness? Headache (2001) 41:629–37. 10.1046/j.1526-4610.2001.041007629.x 11554950

[8] Yu R Gollub RL Spaeth R Napadow V Wasan A Kong J. Disrupted functional connectivity of the periaqueductal gray in chronic low back pain. Neuroimage Clin (2014) 6:100–8. 10.1016/j.nicl.2014.08.019 25379421 PMC4215524

[9] Truini A Tinelli E Gerardi MC Calistri V Iannuccelli C La Cesa S Abnormal resting state functional connectivity of the periaqueductal grey in patients with fibromyalgia. Clin Exp Rheumatol (2016) 34:S129–33. 27157397

[10] Heinricher MM Tavares I Leith JL Lumb BM. Descending control of nociception: specificity, recruitment and plasticity. Brain Res Rev (2009) 60:214–25. 10.1016/j.brainresrev.2008.12.009 19146877 PMC2894733

[11] Lei J Sun T Lumb BM You H-J. Roles of the periaqueductal gray in descending facilitatory and inhibitory controls of intramuscular hypertonic saline induced muscle nociception. Exp Neurol (2014) 257:88–94. 10.1016/j.expneurol.2014.04.019 24792920

[12] Akerman S Holland PR Goadsby PJ. Diencephalic and brainstem mechanisms in migraine. Nat Rev Neurosci (2011) 12:570–84. 10.1038/nrn3057 21931334

[13] Li YQ Shinonaga Y Takada M Mizuno N. Demonstration of axon terminals of projection fibers from the periaqueductal gray onto neurons in the nucleus raphe magnus which send their axons to the trigeminal sensory nuclei. Brain Res (1993) 608:138–40. 10.1016/0006-8993(93)90784-K 7684309

[14] Hoskin KL Bulmer DC Lasalandra M Jonkman A Goadsby PJ. Fos expression in the midbrain periaqueductal grey after trigeminovascular stimulation. J Anat (2001) 198:29–35. 10.1046/j.1469-7580.2001.19810029.x 11215764 PMC1468188

[15] Li YQ Takada M Shinonaga Y Mizuno N. Direct projections from the midbrain periaqueductal gray and the dorsal raphe nucleus to the trigeminal sensory complex in the rat. Neuroscience (1993) 54:431–43. 10.1016/0306-4522(93)90264-G 7687754

[16] Borsook D Burstein R. The enigma of the dorsolateral pons as a migraine generator. Cephalalgia (2012) 32:803–12. 10.1177/0333102412453952 22798640 PMC3711518

[17] Schmitz N Admiraal-Behloul F Arkink EB Kruit MC Schoonman GG Ferrari MD Attack frequency and disease duration as indicators for brain damage in migraine. Headache (2008) 48:1044–55. 10.1111/j.1526-4610.2008.01133.x 18479421

[18] Kruit MC van Buchem MA Hofman PAM Bakkers JTN Terwindt GM Ferrari MD Migraine as a risk factor for subclinical brain lesions. JAMA (2004) 291:427–34. 10.1001/jama.291.4.427 14747499

[19] Xue T Yuan K Zhao L Yu D Zhao L Dong T Intrinsic brain network abnormalities in migraines without aura revealed in resting-state fMRI. PLoS One (2012) 7:e52927. 10.1371/journal.pone.0052927 23285228 PMC3532057

[20] Palm-Meinders IH Koppen H Terwindt GM Launer LJ Konishi J Moonen JME Structural brain changes in migraine. JAMA (2012) 308:1889–97. 10.1001/jama.2012.14276 23150008 PMC3633206

[21] Linnman C Beucke J-C Jensen KB Gollub RL Kong J. Sex similarities and differences in pain-related periaqueductal gray connectivity. Pain (2012) 153:444–54. 10.1016/j.pain.2011.11.006 22154332 PMC3558685

[22] Beck AT Steer RA Brown GK Beck Depression Inventory-II. San Antonio, TX: Psychological Corporation (1996). p. 72498–8204.

[23] Woolrich MW Jbabdi S Patenaude B Chappell M Makni S Behrens T Bayesian analysis of neuroimaging data in FSL. Neuroimage (2009) 45:S173–86. 10.1016/j.neuroimage.2008.10.055 19059349

[24] Woolrich MW Behrens TEJ Beckmann CF Smith SM. Mixture models with adaptive spatial regularization for segmentation with an application to FMRI data. IEEE Trans Med Imaging (2005) 24:1–11. 10.1109/TMI.2004.836545 15638182

[25] Maldjian JA Laurienti PJ Kraft RA Burdette JH. An automated method for neuroanatomic and cytoarchitectonic atlas-based interrogation of fMRI data sets. Neuroimage (2003) 19:1233–9. 10.1016/S1053-8119(03)00169-1 12880848

[26] Inghilleri M Gilio F Conte A Frasca V Marini Bettolo C Iacovelli E Topiramate and cortical excitability in humans: a study with repetitive transcranial magnetic stimulation. Exp Brain Res (2006) 174:667–72. 10.1007/s00221-006-0506-7 16896986

[27] Hodkinson DJ Veggeberg R Kucyi A van Dijk KRA Wilcox SL Scrivani SJ Cortico-cortical connections of primary sensory areas and associated symptoms in migraine. eNeuro (2016) 3(6):e0163–16.2016 10.1523/ENEURO.0163-16.2016 PMC5239993 28101529

[28] Youssef AM Ludwick A Wilcox SL Lebel A Peng K Colon E In child and adult migraineurs the somatosensory cortex stands out … again: an arterial spin labeling investigation. Hum Brain Mapp (2017) 38:4078–87. 10.1002/hbm.23649 28560777 PMC5509164

[29] Tracey I Mantyh PW. The cerebral signature for pain perception and its modulation. Neuron (2007) 55:377–91. 10.1016/j.neuron.2007.07.012 17678852

[30] Zhang L Zhang Y Zhao Z-Q. Anterior cingulate cortex contributes to the descending facilitatory modulation of pain via dorsal reticular nucleus. Eur J Neurosci (2005) 22:1141–8. 10.1111/j.1460-9568.2005.04302.x 16176356

[31] Schweinhardt P Bushnell MC Pain imaging in health and disease – how far have we come? J Clin Invest (2010) 120:3788–97. 10.1172/JCI43498 21041961 PMC2964988

[32] Hadjipavlou G Dunckley P Behrens TE Tracey I. Determining anatomical connectivities between cortical and brainstem pain processing regions in humans: a diffusion tensor imaging study in healthy controls. Pain (2006) 123:169–78. 10.1016/j.pain.2006.02.027 16616418

[33] Vecchia D Pietrobon D. Migraine: a disorder of brain excitatory-inhibitory balance? Trends Neurosci (2012) 35:507–20. 10.1016/j.tins.2012.04.007 22633369

[34] Mainero C Louapre C Migraine and inhibitory system – I can’t hold it! Curr Pain Headache Rep (2014) 18:426 10.1007/s11916-014-0426-3 24824993

[35] Yu C-X Li B Xu Y-K Ji T-T Li L Zhao C-J Altered functional connectivity of the periaqueductal gray in chronic neck and shoulder pain. Neuroreport (2017) 28:720–5. 10.1097/WNR.0000000000000819 28574927

[36] Geddes SD Assadzada S Lemelin D Sokolovski A Bergeron R Haj-Dahmane S Target-specific modulation of the descending prefrontal cortex inputs to the dorsal raphe nucleus by cannabinoids. Proc Natl Acad Sci U S A (2016) 113:5429–34. 10.1073/pnas.1522754113 27114535 PMC4868450

[37] Mainero C Boshyan J Hadjikhani N. Altered functional magnetic resonance imaging resting-state connectivity in periaqueductal gray networks in migraine. Ann Neurol (2011) 70:838–45. 10.1002/ana.22537 22162064 PMC3243965

[38] Boyer N Dallel R Artola A Monconduit L. General trigeminospinal central sensitization and impaired descending pain inhibitory controls contribute to migraine progression. Pain (2014) 155:1196–205. 10.1016/j.pain.2014.03.001 24631586

[39] Lovati C D’Amico D Bertora P Rosa S Suardelli M Mailland E Acute and interictal allodynia in patients with different headache forms: an Italian pilot study. Headache (2008) 48:272–7. 10.1111/j.1526-4610.2007.00998.x 18081821

[40] Weissman-Fogel I Sprecher E Granovsky Y Yarnitsky D. Repeated noxious stimulation of the skin enhances cutaneous pain perception of migraine patients in-between attacks: clinical evidence for continuous sub-threshold increase in membrane excitability of central trigeminovascular neurons. Pain (2003) 104:693–700. 10.1016/S0304-3959(03)00159-3 12927642

[41] de Tommaso M Sciruicchio V. Migraine and central sensitization: clinical features, main comorbidities and therapeutic perspectives. Curr Rheumatol Rev (2016) 12:113–26. 10.2174/1573397112666151231110813 26717950

[42] Burstein R Levy D Jakubowski M. Effects of sensitization of trigeminovascular neurons to triptan therapy during migraine. Rev Neurol (Paris) (2005) 161:658–60. 10.1016/S0035-3787(05)85109-4 16141951

[43] Henssen DJHA Kurt E Kozicz T van Dongen R Bartels RHMA van Cappellen van Walsum A-M. New insights in trigeminal anatomy: a double orofacial tract for nociceptive input. Front Neuroanat (2016) 10:53. 10.3389/fnana.2016.00053 27242449 PMC4861896

[44] Borsook D Aasted CM Burstein R Becerra L. Migraine mistakes: error awareness. Neuroscientist (2014) 20:291–304. 10.1177/1073858413503711 24047609

[45] Nachev P Kennard C Husain M. Functional role of the supplementary and pre-supplementary motor areas. Nat Rev Neurosci (2008) 9:856–69. 10.1038/nrn2478 18843271

[46] Loggia ML Berna C Kim J Cahalan CM Gollub RL Wasan AD Disrupted brain circuitry for pain-related reward/punishment in fibromyalgia. Arthritis Rheumatol (2014) 66:203–12. 10.1002/art.38191 24449585 PMC4516215

[47] Koyama T McHaffie JG Laurienti PJ Coghill RC. The subjective experience of pain: where expectations become reality. Proc Natl Acad Sci U S A (2005) 102:12950–5. 10.1073/pnas.0408576102 16150703 PMC1200254

[48] Salomons TV Johnstone T Backonja M-M Davidson RJ. Perceived controllability modulates the neural response to pain. J Neurosci (2004) 24:7199–203. 10.1523/JNEUROSCI.1315-04.2004 15306654 PMC6729173

[49] Salomons TV Moayedi M Weissman-Fogel I Goldberg MB Freeman BV Tenenbaum HC Perceived helplessness is associated with individual differences in the central motor output system. Eur J Neurosci (2012) 35:1481–7. 10.1111/j.1460-9568.2012.08048.x 22564074

[50] Ge HT Liu HX Xiang J Miao AL Tang L Guan QS Abnormal cortical activation in females with acute migraine: a magnetoencephalography study. Clin Neurophysiol (2015) 126:170–9. 10.1016/j.clinph.2014.03.033 24854723

[51] Yu D Yuan K Zhao L Zhao L Dong M Liu P Regional homogeneity abnormalities in patients with interictal migraine without aura: a resting-state study. NMR Biomed (2012) 25:806–12. 10.1002/nbm.1796 22020869

[52] Rocca MA Colombo B Pagani E Falini A Codella M Scotti G Evidence for cortical functional changes in patients with migraine and white matter abnormalities on conventional and diffusion tensor magnetic resonance imaging. Stroke (2003) 34:665–70. 10.1161/01.STR.0000057977.06681.11 12624289

[53] Barker RA The basal ganglia and pain. Int J Neurosci (1988) 41:29–34. 10.3109/00207458808985739 3045040

[54] Chudler EH Dong WK. The role of the basal ganglia in nociception and pain. Pain (1995) 60:3–38. 10.1016/0304-3959(94)00172-B 7715939

[55] Moulton EA Becerra L Maleki N Pendse G Tully S Hargreaves R Painful heat reveals hyperexcitability of the temporal pole in interictal and ictal migraine states. Cereb Cortex (2011) 21:435–48. 10.1093/cercor/bhq109 20562317 PMC3020583

[56] Maleki N Becerra L Nutile L Pendse G Brawn J Bigal M Migraine attacks the basal ganglia. Mol Pain (2011) 7:71. 10.1186/1744-8069-7-71 21936901 PMC3192678

[57] Chen Z Chen X Liu M Liu S Ma L Yu S. Disrupted functional connectivity of periaqueductal gray subregions in episodic migraine. J Headache Pain (2017) 18:36. 10.1186/s10194-017-0747-9 28321594 PMC5359195

[58] Borsook D Upadhyay J Chudler EH Becerra L A key role of the basal ganglia in pain and analgesia – insights gained through human functional imaging. Mol Pain (2010) 6:27 10.1186/1744-8069-6-27 20465845 PMC2883978

[59] Sillery E Bittar RG Robson MD Behrens TEJ Stein J Aziz TZ Connectivity of the human periventricular-periaqueductal gray region. J Neurosurg (2005) 103:1030–4. 10.3171/jns.2005.103.6.1030 16381189

[60] Rezai AR Lozano AM Crawley AP Joy ML Davis KD Kwan CL Thalamic stimulation and functional magnetic resonance imaging: localization of cortical and subcortical activation with implanted electrodes. Technical note. J Neurosurg (1999) 90:583–90. 10.3171/jns.1999.90.3.0583 10067936

[61] Krout KE Loewy AD. Periaqueductal gray matter projections to midline and intralaminar thalamic nuclei of the rat. J Comp Neurol (2000) 424:111–41. 10.1002/1096-9861(20000814)424:1<111::AID-CNE9>3.0.CO;2-3 10888743

[62] Wu D Wang S Stein JF Aziz TZ Green AL. Reciprocal interactions between the human thalamus and periaqueductal gray may be important for pain perception. Exp Brain Res (2014) 232:527–34. 10.1007/s00221-013-3761-4 24217977

[63] Schwedt TJ Larson-Prior L Coalson RS Nolan T Mar S Ances BM Allodynia and descending pain modulation in migraine: a resting state functional connectivity analysis. Pain Med (2014) 15:154–65. 10.1111/pme.12267 24165094 PMC4188437

[64] Moulton EA Burstein R Tully S Hargreaves R Becerra L Borsook D. Interictal dysfunction of a brainstem descending modulatory center in migraine patients. PLoS One (2008) 3:e3799. 10.1371/journal.pone.0003799 19030105 PMC2582961

[65] Moulton EA Schmahmann JD Becerra L Borsook D. The cerebellum and pain: passive integrator or active participator? Brain Res Rev (2010) 65:14–27. 10.1016/j.brainresrev.2010.05.005 20553761 PMC2943015

[66] Vincent M Hadjikhani N. The cerebellum and migraine. Headache (2007) 47:820–33. 10.1111/j.1526-4610.2006.00715.x 17578530 PMC3761082

[67] Ducros A Denier C Joutel A Vahedi K Michel A Darcel F Recurrence of the T666M calcium channel CACNA1A gene mutation in familial hemiplegic migraine with progressive cerebellar ataxia. Am J Hum Genet (1999) 64:89–98. 10.1086/302192 9915947 PMC1377706

[68] Siegel P Wepsic JG Alteration of nociception by stimulation of cerebellar structures in the monkey. Physiol Behav (1974) 13:189–94. 10.1016/0031-9384(74)90033-X 4212925

[69] Liu FY Qiao JT Dafny N. Cerebellar stimulation modulates thalamic noxious-evoked responses. Brain Res Bull (1993) 30:529–34. 10.1016/0361-9230(93)90079-Q 8457903

[70] Moulton EA Elman I Pendse G Schmahmann J Becerra L Borsook D. Aversion-related circuitry in the cerebellum: responses to noxious heat and unpleasant images. J Neurosci (2011) 31:3795–804. 10.1523/JNEUROSCI.6709-10.2011 21389234 PMC3063442

[71] Mehnert J Schulte L Timmann D May A. Activity and connectivity of the cerebellum in trigeminal nociception. Neuroimage (2017) 150:112–8. 10.1016/j.neuroimage.2017.02.023 28192274

[72] Stoodley CJ Schmahmann JD. Functional topography in the human cerebellum: a meta-analysis of neuroimaging studies. Neuroimage (2009) 44:489–501. 10.1016/j.neuroimage.2008.08.039 18835452

[73] Villemure C Schweinhardt P. Supraspinal pain processing: distinct roles of emotion and attention. Neuroscientist (2010) 16:276–84. 10.1177/1073858409359200 20360603

[74] Roy M Lebuis A Peretz I Rainville P. The modulation of pain by attention and emotion: a dissociation of perceptual and spinal nociceptive processes. Eur J Pain (2011) 15:641.e1–10. 10.1016/j.ejpain.2010.11.013 21196127

[75] Ossipov MH Morimura K Porreca F. Descending pain modulation and chronification of pain. Curr Opin Support Palliat Care (2014) 8:143–51. 10.1097/SPC.0000000000000055 24752199 PMC4301419

[76] Maleki N Becerra L Borsook D. Migraine: maladaptive brain responses to stress. Headache (2012) 52(Suppl 2):102–6. 10.1111/j.1526-4610.2012.02241.x 23030541 PMC3475609

[77] Wacogne C Lacoste JP Guillibert E Hugues FC Le Jeunne C. Stress, anxiety, depression and migraine. Cephalalgia (2003) 23:451–5. 10.1046/j.1468-2982.2003.00550.x 12807524

[78] Dodick DW Review of comorbidities and risk factors for the development of migraine complications (infarct and chronic migraine). Cephalalgia (2009) 29(Suppl 3):7–14. 10.1111/j.1468-2982.2009.02028.x 20017749

[79] Parashar R Bhalla P Rai NK Pakhare A Babbar R. Migraine: is it related to hormonal disturbances or stress? Int J Womens Health (2014) 6:921–5. 10.2147/IJWH.S62922 25368535 PMC4216045

[80] Radat F [Stress and migraine]. Rev Neurol (Paris) (2013) 169:406–12. 10.1016/j.neurol.2012.11.008 23608071

