# Supplementary material for: Responsivity of Periaqueductal Gray Connectivity Is Related to Headache Frequency in Episodic Migraine
Source: Front Neurol. 2018 Feb 13;9:61. doi: 10.3389/fneur.2018.00061 (PMC5816750; doi:10.3389/fneur.2018.00061)
Supplement: Supplementary file 1 [file data_sheet_1.docx]

Supplementary Table 1: The following areas showed a main effect of pain-induced PAG connectivity in both the migraine and control group.

|  |  |  | COG MNI coordinates | | |  |
| --- | --- | --- | --- | --- | --- | --- |
| Brain Region | Side | Z-Stat | X | Y | Z | Vol (voxels) |
| Positive Main Effect |  |  |  |  |  |  |
| WM (cluster over ACC) | L | 3.03 | -16 | 36 | -2 | 1153 |
| Cerebellum Crus I | R | 3.27 | 44 | -64 | -36 | 851 |
| Vermis 8 |  | 3.34 | 0 | -60 | 44 | 526 |
| Fusiform Gyrus | R | 2.69 | 40 | -12 | -38 | 282 |
| Middle Temporal | R | 2.71 | 64 | -32 | 0 | 214 |
| Negative Main Effect |  |  |  |  |  |  |
| Pallidum | L | 2.8 | -20 | -4 | 2 | 863 |
| Lingual Gyrus | R | 2.45 | 22 | -68 | -4 | 653 |
| SMA | R | 2.52 | 8 | 8 | 60 | 375 |
| Sup. Parietal Cortex | L | 2.82 | -14 | -76 | 56 | 276 |
| Mid. Temporal | L | 2.47 | -50 | -20 | 10 | 203 |
| Precentral | L | 2.49 | -42 | 2 | 54 | 176 |
| Cerebellum VIII | R | 2.32 | 6 | -70 | -34 | 152 |
| Postcentral | R | 2.48 | 26 | -46 | 66 | 110 |
| Sup. Parietal Cortex | R | 2.55 | 20 | -72 | 58 | 103 |
| Sup. Frontal | R | 1.89 | 26 | 50 | 38 | 85 |
| Cerebellum VI | R | 2.42 | 32 | -42 | -26 | 68 |
| Mid. Frontal | L | 2.37 | -40 | 44 | 30 | 38 |
| Sup. Occipital | R | 2.37 | 20 | -96 | 22 | 34 |

ACC, Anterior Cingulate Cortex; COG, Center of Gravity; Mid, Middle; MNI, Montreal Neurological Institute coordinates; SMA, Supplementary Motor Area; Sup, Superior; Vol, Volume; WM, White Matter
