# Supplementary material for: Responsivity of Periaqueductal Gray Connectivity Is Related to Headache Frequency in Episodic Migraine
Source: Front Neurol. 2018 Feb 13;9:61. doi: 10.3389/fneur.2018.00061 (PMC5816750; doi:10.3389/fneur.2018.00061)

Positive PAG frequency correlation with subject on topiramate shown on a coronal slice (y = -14) on the left, and without subject on the right:


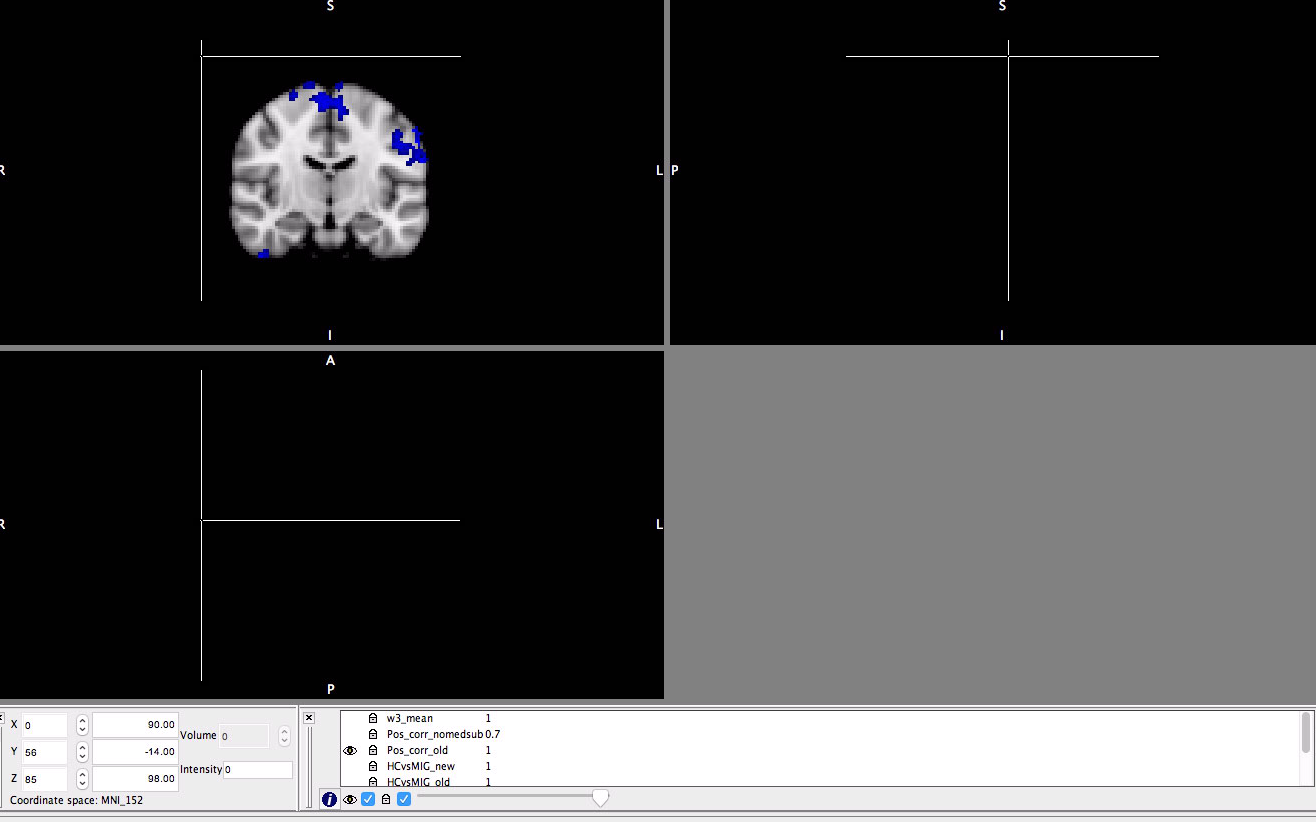

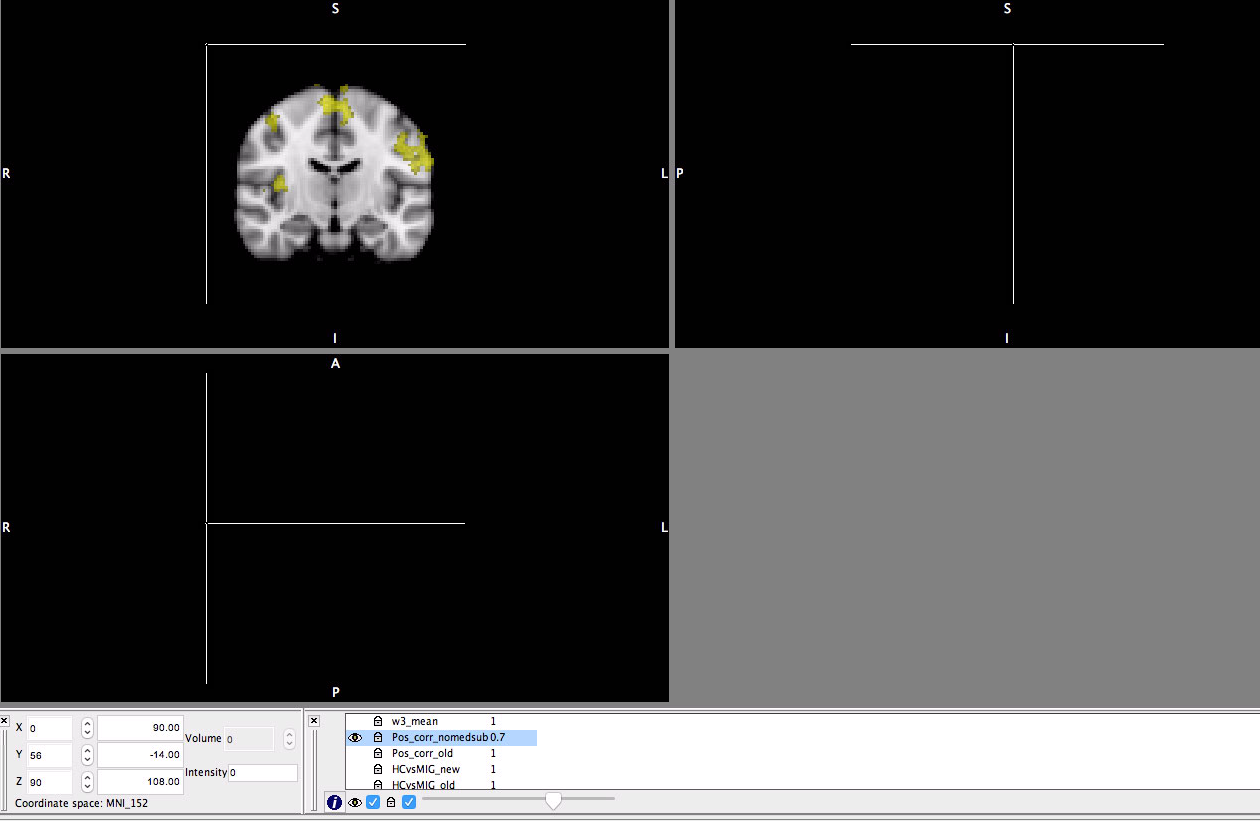


Negative PAG frequency correlation with subject on topiramate shown on an axial slice (z = -4) on the left, and without subject on the right:


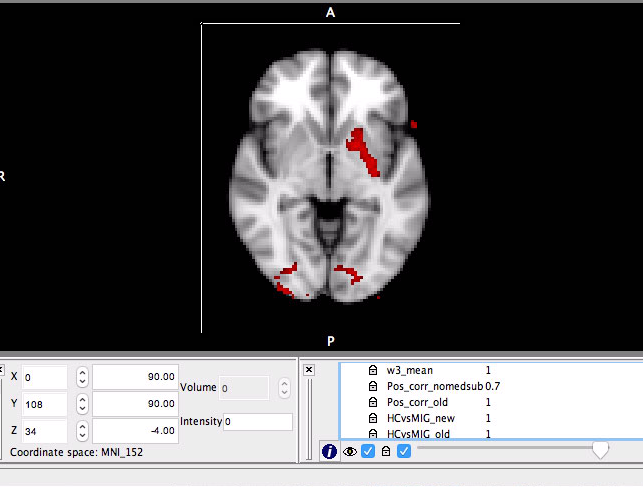

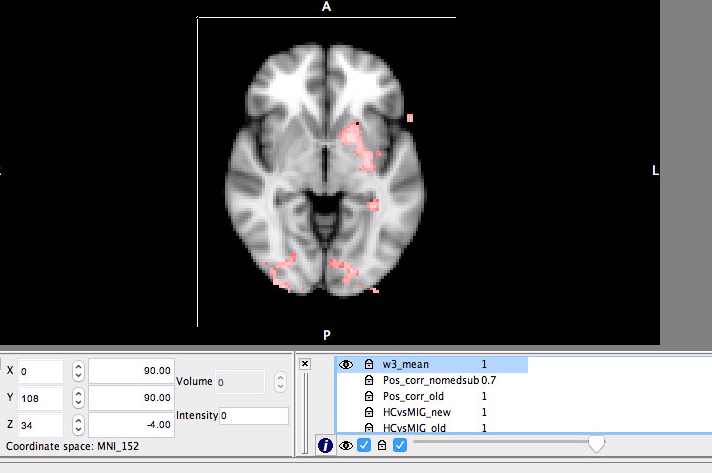

Supplement: Supplementary file 2 [file data_sheet_2.docx]
